# Report of the Medical Department of the Pan-American Exposition, Buffalo, 1901

**Published:** 1902-01

**Authors:** Roswell Park

**Affiliations:** Buffalo, N. Y., Medical Director


### Special Article.

### Report of the Medical Department of the Pan-American Exposition, Buffalo, 1901.

By ROSWELL PARK, M. D., Buffalo, N. Y.,
Medical Director.

THE subjoined report of the workings of The Medical Bureau of the Pan-American Exposition Company is submitted for the information and use of the Board of Directors and all others who may desire to refer to it. This bureau was really organised during midsummer of 1900 by the appointment of the writer as medical director and the recognition of the department as an independent one, the director reporting only to the executive committee and the director-general. The writer was given free power in the selection of his assistants and in operating his department. He was informed that the sum of $20,000 had been appropriated for the expense of the department from its inception to the close of the exposition. Out of this, the hospital was to be equipped and all the running expenses paid.

Organisation being the first requirement, he selected Dr. Vertner Kenerson as deputy medical director, Miss Adella Walters, a graduate of the Buffalo General Hospital, as superintendent, and Miss Minnie A. VanEvery, who was to act in combined capacity of stenographer and trained nurse. About the 1st of September, 1900, four rooms in the Service Building were assigned for the use of the department. It was not long before it appeared necessary to have a resident physician who should be available for service at all hours of the night and day, since more or less night work was going on, and there was always a liability to accident. Accordingly Dr. Alexander Allan was selected and took up his residence in the Service Building a couple of weeks later, Dr. Eli Schriver, Jr., substituting for him for a short time. From the 1st of September, 1900, until the 1st of May, 1901, these rooms were made to suffice for the purpose. Most of the cases attended during this time were of the nature of minor injuries, there being occasional cases of illness, and very few serious accidents. During this construction period, 750 people received "first aid" attention in these temporary apartments.

As the season for opening the exposition grew nearer, more detailed plans for caring for visitors were required. Accordingly, a small area between the Service Building and the West Amherst gate was selected as an appropriate site for the hospital. It was intentionally placed near the gate in order that it might be more easily approached by carriages and ambulances from without, as also because there was ready access to the street cars. Plans for the hospital building were prepared by the medical director and Mr. Weatherwax, the chief draughtsman in the Service Building. These provided for three wards, two for men and one for women, with accommodations for 25 beds, with a complete operating room outfit, suitable offices, accommodations for nurses and resident physicians, and sheds for ambulances. Descriptions of this building have already been published and it is perhaps hardly necessary here to go into detail.[1] Suffice it to say that a practically complete small hospital was erected and equipped with everything needed for the purpose. A part of the purely surgical equipment was loaned by The Jeffrey-Fell Company, of this city, as a part of their exhibit, and to them this department is under many obligations for courtesies and assistance of this kind.

The Wagner Company also supplied, in the same way, a large static machine, with an *x*-ray outfit, and numerous smaller exhibits were sent in for use by various individuals and firms. Operating table, ordinary sterilising appliances and instruments were provided at the outset by the Exposition Company.

The department force and physicians was now increased by several important appointments. Dr. Nelson W. Wilson was appointed sanitary officer, and at once entered upon an exceedingly active campaign, which resulted in a sanitary condition of affairs which was noticed by almost every visitor to the Exposition. More will be said about these sanitary arrangements below.

During the first month of the Exposition period, the roster of the hospital was as follows: Dr. Roswell Park, medical director; Dr. Vertner Kenerson, deputy medical director; Dr. Nelson W. Wilson, sanitary inspector; Miss Adella Walters, superintendent; Miss Minnie A. VanEvery, stenographer and trained nurse; Drs. A. F. Zittel, Alexander Allan, Geo. McK. Hall and E. C. Mann, house staff; Messrs. B. J. Bixby, Bert Simpson, T. F. Ellis and Norman S. Betts, ambulance men and male nurses.

The May nurses were: Mrs. Laura Hesselberg, Presbyterian Hospital, New York; Miss Claribel Lichtenstein, La Tour Infirmary, New Orleans, La.; Miss Cecil Dodge, Chicago Baptist Hospital, Chicago, Ills.; and Miss Margaret Haines, Buffalo Children's and Woman's Hospitals, Buffalo, N.Y.

---

1. See BUFFALO MEDICAL JOURNAL, April, 1901, p 701.

June nurses: Misses A. L. Greenwood and Miss Florence Hamilton, Buffalo General Hospital; Miss Laura Jarvis, Arnot Ogden Hospital, Elmira, N.Y.; Miss Eleanor Alexander, Kingston, Ont., and Miss Mabel Farnsworth, Buffalo.

July nurses: Miss M. McCulloch, Lansing Hospital, Lansing, Mich ; Miss Jennie Wanner, Garfield Memorial Hospital, Washington, D.C.; Miss Maud Trueman, Royal Victoria Hospital, Barrie, Ont.; Miss Hattie Cary, Buffalo General Hospital; Mrs. Eunice Hughes, University of Maryland Hospital; and Miss Dodds, Lexington Hospital, Buffalo.

August nurses: Mrs. Anna Richmond, Hants Infirmary, London, Eng.; Miss B. Jelly, London, Ont., General Hospital; Miss Teresa Bartle, St. Luke's Hospital, Chicago, Ill.; Miss J. B. Downing and Miss Katherine Simmons, Roosevelt Hospital, New York, and Miss Mae Railton, Buffalo General Hospital.

September nurses: Miss K. Simmons, (2d month) in charge of Crèche, Roosevelt Hospital, New York; Miss Dorchester, Buffalo General Hospital; Miss Baron, Long Island College Hospital, Brooklyn, N. Y.; Miss Shannon, Spanish War Nurse and General Memorial Hospital, New York; and Miss Morris, St. Luke's Hospital, New York.

October nurses: Miss M. L. Davidson, Long Island College Hospital, Brooklyn, N. Y.; Miss Anna Hadden, Orange (N. J.) Training School, Orange, N. J.; Miss Charity Babcock, Johns Hopkins Hospital, Baltimore, Md.; Miss M. Michell, Jewish Hospital, Cincinnati, O.; and Misses M. Conners and McLaren, Rochester City Hospital.

Arrangements had been perfected during the preceding months for ample supply of nurses, as well as for their selection from various schools in the country. A circular of information was prepared and sent out, inviting applications and informing applicants that their applications for one month tour of duty would be received, and that for these services they would be given all the privileges of the fair, with ample opportunity off duty to see all that was to be seen, and that they would receive also $25 each as a mere honorarium intended to cover their traveling expenses, and the like, in addition to their board and laundry. Over 150 requests from all parts of the country were received. From these selection was made, as above, those serving for each month being indicated. A housemaid was also added and served in the diet kitchen.

The rule was laid down positively, first of all, that there was to be no charge for services rendered, and that these were to be only of the first aid order, patients being kept only long enough to enable them to leave the building or the grounds in suitable condition. The rule was also made that no patients were to be kept over night, but were to be sent to their homes, to the railroad stations, or to some other hospital, as they might elect or the circumstances might require. This rule was disregarded only in one or two instances in the case of some of the resident population who had no place to go, or who could not be accompanied by an interpreter.

Suitable badges were provided for the three principal medical officers, of gold or silver, and for all the hospital corps of silver, with the red cross as a prominent feature, by which their wearers were enabled to pass, at all times, to all points within the grounds. All the male members of the hospital corps were dressed in suitable blue uniforms for cold weather, and white for warm weather, and every nurse when on duty wore the uniform of the school from which she graduated.

The Exposition guards were instructed to recognise the badges of the medical corps and to render such assistance and coöperation as might be called for at any time. On numerous occasions they were of no small assistance.

The Medical Department was given jurisdiction over all the sanitary arrangements of the grounds and matters pertaining to public health. This included supervision of the houses of public comfort, all the sewerage and drainage arrangements of the grounds themselves, the foods served in all the restaurants, the soft drinks in the various restaurants and concessions, and everything that pertained to public health. How carefully these sanitary arrangements were attended to will be evidenced by the fact that few, if any, cases of illness due to lack of any precautions have ever come to our knowledge. Not a few persons suffered from over-indulgence, but so far we have learned no one suffered from improper character of food, save in the instances, to be referred to below, of the Indian children who were made sick by eating decayed fruit thrown out from the Horticultural Building.

The hospital building was placed in perfect telephonic communication with all parts of the grounds and nearly all of the ambulance calls which came in were reported over the telephone. A pay roll was sent in every month, having upon it the name of every employee of the department. No one was asked to do work for nothing, and every one was paid for his or her work exactly as per the original agreement.

Early in the progress of the fair it had been suggested to establish a Crèche. By resolution of the directors this was not done, however, until August, when large tents were erected, cots provided, and one of our nurses detailed for this especial work. A nursery maid was also employed. For the care of children in the Crèche a nominal charge was made of 25 to 50 cents, according to the time they remained, and these were the only fees or money accepted by the department during its entire service. The Crèche proved to be very popular, and during the hot weather of August and early September, was well patronised. It a little more than covered the actual outlay it required.

The original intention was to have three ambulances, one of each type,—electric, gasoline and steam. It was found, however, that none of the manufacturers using gasoline engines made an ambulance, and the Rochester firm which promised a steam motor vehicle failed to keep their agreement. Consequently, the only ambulance maintained upon the grounds throughout the season for our work was that on the Riker system, furnished by The New York Electric Motor Vehicle Company. This was put in commission before the opening of the fair as an exhibit, and, during the season, made many hundreds of trips of various lengths. Not once did it disappoint us in its reliability and constant availability, and while I feel that we are under very many obligations to the company which furnished it, I cannot speak in too high terms of its efficiency.

The water supply to the grounds and buildings was ample and always reliable. The water supply of the City of Buffalo being at all times exceptionally good, we had but very little to contend with in the matter of pollution from this source. Several attempts were made to create newspaper sensations at a distance from Buffalo, and to make it appear that exposition visitors had contracted typhoid upon our grounds. Every one of these, however, when investigated, proved to be purely sensational and without foundation.

The sewerage of the grounds was also excellent, and we had very few complaints on this account. We were met more than half way by the city authorities whenever it was necessary, and I may say at this point that there was throughout the whole period the heartiest coöperation between the City Health Department, with its various officials, and ourselves. Health Commissioner Wende extended many courtesies to us in this regard.

A very complete set of hospital records was kept. In a large book were entered the name, age, temporary and permanent addresses of every patient, along with the nature of the trouble for which he came to the hospital, and also the final disposition of the case. Aside from this, a duplicate card record was kept, which included the number, as taken from our large book, the name, residence, the city address, hour and date of admission and of discharge, the name of the employee, if the patient were an employee, the method of his admission, *i.e.*, whether he came by ambulance, rolling chair, stretcher or on foot, the moment when the ambulance call was received, and the moment when the patient was delivered at the hospital, diagnosis, brief note of treatment, and a statement regarding the final disposition of the patient. One set of these cards was filed for our own reference and use and the other was sent daily to the Committee on Law and Insurance, so that every night the legal authorities of the Exposition knew just what accidents had been cared for, or possible liabilities incurred through the day. This was an extremely valuable feature of our work and saved much trouble. Our own card records were preserved in alphabetical order, as well.

From the outset, I insisted that patients coming to us for help should be treated with every courtesy and with becoming privacy. During the earlier weeks of the fair, I had considerable trouble with reporters from various papers who exhibited an undue and unbecoming anxiety to get what they considered news and construct out of the items given them more or less sensational reports. The rule was laid down, and strictly adhered to, that no names should be given out without the consent of the individuals concerned, that our records were private, that our hospital was a retreat and an asylum to which those who were sick and injured could come when they desired for refuge from the public, and that from this rule there should be no departure. The local manager of the local press made himself peculiarly offensive in protesting against this rule, but it was nevertheless adhered to, and the riot act was read to him on more than one occasion.

Herewith are submitted statistical tables, from which sufficient information may be gleaned as to the general character of our work, number of cases, etc. The tables will be self explanatory and probably need no further comment:

TABLE I.

CLASSIFIED LIST OF CASES NOS. 1 TO 5,573.

| | | |
|---|---|---|
| Continued and eruptive fevers | | 17 |
| Malaria | | 7 |
| Rheumatism | | 75 |
| Diseases of nervous system | | 162 |
| Diseases of circulatory system | | 189 |
| Diseases of respiratory system | | 223 |
| Diseases of digestive system | | 1,971 |
| Diseases of lymphatic system | | 14 |
| Diseases of urinary system | | 20 |
| Diseases of generative system (male) | | 15 |
| Diseases of generative system (female) | | 45 |
| Diseases of skin | | 111 |
| Diseases of and injuries to the eye | | 247 |
| Diseases of ear | | 28 |
| Diseases of chest | | 12 |
| Diseases of throat | | 205 |
| Heat exhaustion | | 55 |
| Exhaustion (other) | | 51 |
| Syphilis | | 5 |
| Burns and scalds | | 97 |
| Minor injuries and wounds | | 1,482 |
| Scalp wounds | | 80 |
| Sprains | | 158 |
| Dislocations | | 5 |
| Gunshot wounds | | 6 |
| Electric shock | | 3 |
| Intoxication | | 4 |
| Toothache | | 161 |
| Teeth extracted | | 43 |
| Fractures | | 78 |
| Fracture of radius | 4 | |
| Fracture of clavicle | 2 | |
| Fracture of fingers | 7 | |
| Fracture of nose | 7 | |
| Fracture of arm | 11 | |
| Fracture of skull | 4 | |
| Fracture of leg | 7 | |
| Fracture of foot | 2 | |
| Fracture of ribs | 6 | |
| Fracture, Potts's | 1 | |
| Fracture of toes | 3 | |
| Fracture of femur | 2—78 | |
| Deaths in Pan-American hospital | | 4 |
| | | 5,573 |

### TABLE II.

### DEATHS IN PAN-AMERICAN HOSPITAL AND AMBULANCE.

Case No. 2,226. Pneumonia.
Case No. 4,175. Apoplexy.
Case No. 5,557. Heart Disease.
Death in ambulance of man shot during a fracas in the Free Midway.
Baby in Indian Village, died of entero-colitis, treatment being refused.
Premature birth (6 or 7 mos.) in African Village.
Indian baby died, in hospital, of inspiration pneumonia, following inspiration of some grains of partially cooked rice.

### DEATHS BY ACCIDENT ON THE GROUNDS.

One man killed by cars before Medical Bureau was organised.
Case No. 613. Struck by Belt Line train; both legs severed from body. Killed instantly.
Case No. 631. Fracture of skull. Killed instantly.
Case No. 3,490. Bullet through sternum. Killed instantly.
Case No. 3,568. Fracture of skull. Killed instantly.
Two men killed by electricity. Not taken to hospital.

### TABLE III.

### BIRTHS.

Two births in Indian Village. One birth in Filipino Village.

### TABLE IV.

### TOTALS OF PATIENTS BY MONTHS.

| | | | | | | |
|---|---|---|---|---|---|---|
| August, | 1900 | 17 | April, | 1901 | 167 | |
| September, | " | 60 | May, | " | 482 | |
| October | " | 86 | June, | " | 660 | |
| November, | " | 94 | July | " | 1,018 | |
| December, | " | 73 | August, | " | 1,145 | |
| January, | 1901 | 92 | September, | " | 954 | |
| February, | " | 59 | October, | " | 554 | |
| March, | " | 100 | | | | 5,561 |

| | | |
|---|---|---|
| Total for construction period up to May 1, 1901 | 748 | |
| Total for Exposition period up to November 1, 1901 | 4,813 | |
| | | 5,561 |
| Daily average for construction period | | 2 |
| Daily average for Exposition period | | 26 |

### TABLE V.

### NUMBER OF PATIENTS SENT TO EACH HOSPITAL.

| | | | | |
|---|---|---|---|---|
| Buffalo General | 36 | Homeopathic | 8 | |
| German | 2 | Riverside | 2 | |
| German Deaconess' | 1 | Sisters' | 21 | |
| | | | | 70 |

SUMMARY.

| | | |
|---|---|---|
| Total number of diagnoses . . . . . . . . . . . . . . . | 5,572 | |
| Total number of persons killed . . . . . . . . . . . . | 4 | |
| | | 5,576 |
| Total number of patients treated . . . . . . . . . . . . | 5,567 | |
| Total number of cases with more than one major injury . . | 9 | |
| | | 5,576 |

Further details regarding the sanitary inspection of the grounds may be of interest. Much of what follows I have epitomized from the monthly reports of Dr. Wilson, the sanitary inspector. During the earlier part of the fair period two daily inspection tours of the Midway and one of the grounds and buildings were made. Suitable blanks were prepared and sent in to medical headquarters each day, reporting the extent and time of inspection and the results. One of these blanks showed, at a glance, the daily condition of the Exposition; another gave a list of the places inspected each day; another was served on exhibitors and concessionaires on whose premises any sanitary nuisance existed. In this last those at fault were given a certain number of hours for the correction of the nuisance, while failure on their part to comply with the order within the time limit resulted in the work being done under the supervision of the sanitary officer and the expense charged against the premises. At the conclusion of the necessary work, reinspection was made, and another blank filled in with details and sent to the medical director. During the month of May, for instance, 68 nuisance notices were served, and in only one instance was it necessary to call upon the Superintendent of the Exposition Street Cleaning Department to remedy the fault. Some trouble was met with in that portion of the grounds occupied by the Indian Congress, where the land lay low and where the Indians lived in tepees, which, after the heavy rains of the month, once or twice became almost uninhabitable. In this case the concessionaires voluntarily raised the ground, and the sanitary bureau provided an extra drain, and thus the fault was remedied.

Early in May, or very shortly after their arrival, the Esquimaux in the Esquimau Village developed an epidemic of measles, of which there were, in all, eleven cases. The village was quarantined and a guard stationed at the gate. The cases were transferred, as they occurred, to the contagious pavilion of the General Hospital, and quarantine was maintained until May 30. Mumps also appeared in the Hawaiian Village, but prompt quarantine checked a spread of the disease.

An important part of the work was the regulation and supervision of the milk and cream supply. In every place on the grounds where these articles were on sale the source of supply was learned, and it was ordered that neither of them should be bought from any source save a dealer subject to the City Health Department regulations. When concessionaires did not live up to these requirements, delivery of goods was stopped. Careful inspection of meat and ice boxes and supplies of this character was practised from the beginning, and not a few times bad ice was condemned. Some difficulty was met with in the beginning in ensuring proper cleaning of the tumblers at many of the stands where soft drinks were sold. This was mainly due to the lack of running water at these points.

Every water closet for men was in charge of a male attendant, and a woman was placed in charge of each woman's toilet room. Directions were given which preserved almost absolute cleanliness, and throughout the season there was very little ground for complaint on this score. By the first of June, there were in operation on the grounds over 500 closets and urinals, a map showing the former being kept constantly on file at the Medical Director's office. As warm weather approached, every restaurant was required to furnish the name of the dealers supplying milk, cream, ice cream and meats, and the ice boxes and refrigerators were carefully watched.

In July it became necessary to keep careful watch on the Indian Congress because of the susceptibility of the Indians to tuberculosis. During this month eight were returned to their reservations and the quarters occupied by them thoroughly cleaned and disinfected. In the Filipino and African Villages it became necessary to put up health notices in more than one language, and to issue very positive instructions to managers and interpreters, especially regarding the proper use of ordinary toilet and sanitary provisions. Our principal trouble was to maintain reasonable cleanliness among the inhabitants of The Beautiful Orient. Only after continuous and constant pressure was it possible to get them to keep their stables and yards reasonably clean. More than once it was necessary to seize rotten fruit sold at the fruit stands in the Streets of Cairo. The inhabitants of this concession seemed to be absolutely incorrigible, and incapable of appreciating the ordinary laws of health.

During the month of July, there were 29 restaurants open and doing business, and 43 stands at which soft drinks were sold.

The resident population for the month was—people, 1,535; animals, 607. Food supplies were frequently inspected on receipt, but about the only time when it was necessary to order food thrown out was one occasion in August at a restaurant in the Beautiful Orient. About the only influence which could be effectually brought to bear on the people in this concession was a threat to close up the business. The other concessions gave very little trouble. Once at the Filipino Village an old woman well advanced with pulmonary tuberculosis was found at work stripping tobacco for the cigar makers. She was promptly removed from the tobacco room and directions given regarding the disinfection of her sputum. During this month of August also an Indian baby died at the hospital of inspiration pneumonia, which was not due to exposure, but to trouble following inspiration into the lungs of some grains of partially cooked rice.

By the end of July there were 36 restaurants and eating places, 14 kitchens on concessions and villages, and 57 soft drink stands, and the resident population had increased to 1652. That the residents of the concessions had adopted stricter sanitary measures was evident from the fact that during this month it was necessary to issue only 43 time-limit notices threatening condemnation for failure to correct. After a while, the regular hours of inspection were abandoned and the inspections were made at irregular and intentionally unexpected intervals in order that no preparation for them could be made. The results of this change were an evident improvement in all sanitary conditions. In no case during August was it necessary to condemn milk or cream, and only one eating place gave any serious trouble. This was the Nebraska Sod House, which was an almost constant source of bother and which later had to be closed. Night inspections revealed the fact that many people were in the habit of sleeping beneath the counters in booths in various streets. The practice was stopped, for instance, of making of candy in a booth in which a family of four lived, cooked, ate and slept.

On the 9th of August an Indian child was taken seriously ill. Treatment by the medical department was refused and the Indian medicine man took charge of the case. The child died within twenty-four hours. Investigation showed that the death of this child and the serious illness of two others were due to the fact that the Indian children were in the habit of eating fruit which their parents had secured from the Horticultural Building, which fruit was discarded because it was no longer fresh nor presentable. It was hard to impress the Indian parents with the idea that this trouble was due not to the "anger of the Great Spirit," but to disregard of common hygienic laws. Dr. Wilson, however, succeeded in impressing them with the idea that it was rotten fruit which "the Great Spirit" did not like to have them eat. During this month also four more Indians were returned to their reservations on account of tuberculosis.

A mild epidemic of barber's itch appeared upon the grounds, which was traced to barber shops outside, where a number of guards and attendants were in the habit of being shaved. Suitable treatment of individuals and suitable instructions at the barber shops soon checked this difficulty. By the end of August the number of people living on the grounds was 1703, and the number of animals 1,255.

A little ripple of excitement was caused by newspaper statements, that eight persons coming from Newark, N.J., had contracted typhoid fever at the Exposition. It appeared, on careful examination, that typhoid was prevalent in Newark at the time these people left home, and it was easy to see that their typhoid was contracted before they came to Buffalo, although it may not have appeared until after their return home.

In September, national calamity led to the closing of the Exposition on three different days. It was naturally expected that there would be temptation to keep food supplies too long and it became necessary to condemn a number of supplies of meat and other food, as well as of milk and cream. Every restaurant and every kitchen was carefully inspected prior to reopening and where there was any doubt condemnation was enforced.

The Beautiful Orient continued to give more or less trouble, and on one evening visit twelve people were found occupying one small room. It was cleared and disinfected, and the practise prohibited. It became necessary to return three more Indians to their reservations, one had a broken arm, the others were suffering from tuberculosis. A mild outbreak of measles occurred in the camp of the United States Marines. The cases were sent to the hospital and tents and quarters were thoroughly fumigated. During September a few cases of milk fever were reported among the animals of the Live Stock exhibit. They were properly cared for by a veterinarian, and the disease did not spread. During September, the largest resident population was met with, namely, 1792.

During the closing months of the fair there seemed to be temptation to carelessness on the part of some of the concessionaires and vigilance was redoubled. While it was necessary in a number of instances to condemn food during this month, there was but one known instance of serious trouble which might have been charged to negligence in this respect. This was the case of a young woman who was taken acutely sick on the grounds, and who died later at the General Hospital. A careful investigation showed that she ate little or nothing upon the grounds, and that, although she seemed to die of ptomaine poisoning, it was not due to anything which she had procured within the Exposition limits. During October, four more Indians were sent home. At the conclusion of the Exposition period—namely, on the second day of November, all sanitary supervision of the grounds, and the like, was formally transferred to the City Health Department, and the operation of this bureau, in this and all other respects, were completely suspended.

The expenses of the Medical Bureau ran up to a total of $16,332.72. This included all the moneys which were paid out for any purposes connected with the bureau, household equipment, drugs, running expenses, and Créche. This amount includes $4,832.80 expended previous to May 1, and $11,499.92 expended between May 1 and the close of the Exposition. The cost of the hospital was $6,966.54.

A brief study of the weather conditions during the Exposition period may be of interest at this point. It is made up from the records of the Weather Bureau. The mean temperature of May, 1901, was 54°, the coldest in four years, while the precipitation was 3.28, which made it the wettest May in seven years. In June, the mean temperature was 54°, which made it the coldest June in the history of the Weather Bureau in 30 years, while the precipitation was that for the preceding month, making it the wettest June in seven years. From this there was an abrupt change to reverse conditions. The mean temperature for July was 74°, which was only equalled during the past 30 years by two other Julies, while the precipitation was 3.05. August also was warm, but with a light rainfall, which made altogether a beautiful month. September was quite wet during the middle of the month, and its temperature seemed above normal. October proved to be the coldest October in five years, with a mean temperature of 53.° During the fair period of 1901, there were 68 wet days. During the previous year and the same period there were 59 wet days. Briefly summed up, the Exposition furnished us with weather to which quite appropriately Mr. Cuthbertson has applied the terms "rainy, raw, chilly, hot, cold and cloudy", with a notable infrequency of clear days, all which had its effect in discouraging and disheartening would-be visitors to the fair.

If it were the place, a number of things might be said pertaining to the humorous side of the situation. People persisted in mistaking us for a free drug store and trying to get all sorts of prescriptions filled without expense to themselves. They also came in on all sorts of absurd errands, even occasionally soliciting baths. One night when I was at the hospital a negro came in from "Darkest Africa," and, with the utmost seriousness, demanded a dose of poison. As nearly as we could gather from him, this was intended for one of his confrères in the Negro Village with whom he had had a quarrel, and he announced that it was his intention to give him poison rather than club him or stab him, because he thought a natural sort of death would attract less attention than a more conspicuous homicidal attempt.

Of course, by all means the most important work of the medical bureau was connected with the Presidential tragedy, about which so much has already been said that it does not seem necessary to more than allude to it here as part of the work of the bureau. Had it not been for the existence upon the grounds of such a hospital as we had, ready for any such event, it would not have been possible to give the President the advantage of such prompt intervention as that which was afforded. As it was, I have often repeated the remark, which I made during that sad week to numerous members of the government, that "because of what we had provided upon the grounds *the President was not deprived of the benefits of private citizenship*," as he would have been under most other circumstances. The existence of the hospital and the workings of the medical bureau were certainly justified at this time beyond all expectations or desire.

I ought not to close this report of the workings of the Medical Department without, both gracefully and gratefully, acknowledgments, first to the Directors, the Director-General and the Director of Works, to whom I never appealed in vain, and, secondly, to my assistants and staff of helpers, all of whom rendered most cheerful and most effective assistance. Dr. Kenerson was of greatest service, especially during the organisation period, when his readiness and skill in managing details appeared to best possible advantage. To him and to Dr. Wilson, whose services I have already mentioned, I feel very greatly indebted. To the members of the hospital staff, especially to Dr. Zittel, I feel that very much credit is due, and to the nursing staff who served throughout with zeal and fidelity, I feel particularly grateful. For over a year Miss Walters managed the details of the nursing, as well as of household cares, with rare tact and faithfulness, and it would be unfair not to comment most favorably at this point on her work. One and all, however, assisted, each to the best of his or her ability, and the result was a department of which, as the Director-General said, there had been and could be no complaint.

510 DELAWARE AVENUE.

